# Identification of Cytochrome P450 2E1 as a Novel Target in Glioma and Development of Its Inhibitor as an Anti‐Tumor Agent

**DOI:** 10.1002/advs.202301096

**Published:** 2023-06-07

**Authors:** Guiming Hu, Yan Fang, Haiwei Xu, Guanzhe Wang, Rui Yang, Fei Gao, Qingda Wei, Yuhan Gu, Cunzhen Zhang, Jinhuan Qiu, Na Gao, Qiang Wen, Hailing Qiao

**Affiliations:** ^1^ Institute of Clinical Pharmacology Zhengzhou University Kexue Road Zhengzhou 450001 China; ^2^ Department of Pathology The Second Affiliated Hospital of Zhengzhou University Jingba Road Zhengzhou 450014 China; ^3^ School of Pharmaceutical Sciences Zhengzhou University Kexue Road Zhengzhou 450001 China

**Keywords:** CYP2E1, glioma, inflammation, microglia/macrophage, reprogramming, target, tumor microenvironment

## Abstract

Glioblastoma (GBM) is a devastating inflammation‐related cancer for which novel therapeutic targets are urgently required. Previous studies of the authors indicate Cytochrome P450 2E1 (CYP2E1) as a novel inflammatory target and develop a specific inhibitor Q11. Here it is demonstrated that CYP2E1 overexpression is closely related to higher malignancy in GBM patients. CYP2E1 activity is positively correlated with tumor weight in GBM rats. Significantly higher CYP2E1 expression accompanied by increased inflammation is detected in a mouse GBM model. Q11, 1‐(4‐methyl‐5‐thialzolyl) ethenone, a newly developed specific inhibitor of CYP2E1 here remarkably attenuates tumor growth and prolongs survival in vivo. Q11 does not directly affect tumor cells but blocks the tumor‐promoting effect of microglia/macrophage (M/M*φ*) in the tumor microenvironment through PPAR*γ*‐mediated activation of the STAT‐1 and NF‐*κ*B pathways and inhibition of the STAT‐3 and STAT‐6 pathways. The effectiveness and safety of targeting CYP2E1 in GBM are further supported by studies with *Cyp2e1* knockout rodents. In conclusion, a pro‐GBM mechanism in which CYP2E1‐PPAR*γ*‐STAT‐1/NF‐*κ*B/STAT‐3/STAT‐6 axis fueled tumorigenesis by reprogramming M/M*φ* and Q11 as a promising anti‐inflammatory agent for GBM treatment is uncovered.

## Graphical abstract

CYP2E1 increases in glioblastoma and is associated with poor prognosis. A pro‐GBM mechanism in which CYP2E1‐PPAR*γ*‐STAT‐1/NF‐*κ*B/STAT‐3/STAT‐6 axis fueled tumorigenesis by reprogramming M/M*φ* and a newly developed CYP2E1 inhibitor, Q11, as a promising anti‐inflammatory agent for glioblastoma treatment is uncovered.

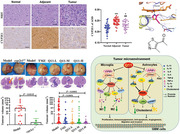

## 1 Introduction

Glioblastoma (GBM) is the most common and lethal subtype of glioma with a median overall survival of 14.6 months^[^ ^1^ ^]^ and a 5‐year survival rate of less than 5%.^[^ ^2^, ^3^ ^]^ Despite the standard treatment of combination of temozolomide (TMZ) and radiation after complete resection of the tumor mass, all GBM eventually acquire therapeutic resistance and progress, with recurrence within six months.^[^ ^4^ ^]^ Due to the high heterogeneity of tumor cells, the etiology and pathophysiologic mechanisms of GBM remain unclear, resulting in limited treatment options. Therefore, it is urgent to explore the pathogenesis and seek new treatments for GBM.

Inflammation in the tumor microenvironment (TME), as a hallmark of cancer^[^ ^5^, ^6^ ^]^ has long been considered to play a crucial role in the initiation and progression of various tumors.^[^ ^7^, ^10^ ^]^ It is now becoming clear that inflammation is an indispensable participant in the neoplastic process, including fostering proliferation, survival, and migration.^[^ ^9^ ^]^ Therefore, the targeting of inflammation represents an attractive strategy both for cancer prevention and therapy.^[^ ^11^ ^]^ Accordingly, anti‐tumor therapies through targeting the TME have made great progress in recent years, with the angiogenesis inhibitor bevacizumab^[^ ^12^, ^13^ ^]^ and immune checkpoint inhibitors PD‐1 and PDL‐1.^[^ ^14^, ^20^ ^]^ Mounting evidence from epidemiological and preclinical studies have shown that inflammation is closely related to GBM.^[^ ^21^, ^22^ ^]^ For example, regular use of nonsteroidal anti‐inflammatory drugs (NSAIDs) is correlated with reduced incidence of GBM.^[^ ^23^ ^]^ Furthermore, a number of cell culture and xenograft studies support the hypothesis that targeting these inflammatory mediators could be beneficial in controlling the insidious behavior of GBM.^[^ ^24^, ^25^ ^]^ This evidence implies that inflammation is involved in the development and progression of GBM and anti‐inflammatory agents could offer a new and possibly improved approach to GBM therapy. Unfortunately, the current anti‐inflammatory therapies have only limited efficacy on GBM, which might be attributed to our incomplete understanding of the mechanisms and of undiscovered inflammatory targets in the pathogenesis of GBM. Hence, in GBM it is worth considering novel inflammatory targets other than cyclooxygenase (COX),^[^ ^26^ ^]^ a target of traditional anti‐inflammatory drugs.

Cytochrome P450 2E1 (CYP2E1), known to metabolize many xenobiotics including alcohol and analgesic drugs and to produce reactive oxygen species (ROS) and reactive metabolites, has been recognized to be involved in inflammatory and oxidative stress processes, which damage DNA, protein, and lipid membranes, and subsequently causing organ damage.^[^ ^27^, ^30^ ^]^ Recently, it is demonstrated that CYP2E1 activity increased significantly in hepatocellular carcinoma^[^ ^31^, ^34^ ^]^ and ovarian cancer^[^ ^35^ ^]^ and correlated to tumor‐associated inflammation, suggesting an important role of CYP2E1 in the development and progression of tumors. It was shown that CYP2E1 played an important role in loss of p53 and p21,^[^ ^36^ ^]^ the tumor suppressor genes for glioma, since we have hypothesized that inhibition of CYP2E1 might inhibit glioma growth. Although many efforts have been devoted to developing CYP2E1 inhibitors,^[^ ^37^, ^39^ ^]^ they have not been successful and have not been applied in clinical practice. Therefore, it is of particular interest to develop CYP2E1 inhibitors with high efficacy and safety.

Our previous study showed that the activity of CYP2E1 was increased in peritumoral tissues of hepatocellular carcinoma (HCC), and inhibition of CYP2E1 could significantly inhibit the occurrence and development of HCC.^[^ ^31^, ^34^ ^]^ In the present study, we investigated the expression of CYP2E1 in peritumoral cells of glioma and studied its relationship with glioma occurrence and progression. CYP2E1 as a new target for glioma anti‐inflammatory therapy was validated and the molecular mechanism of CYP2E1 in the tumorigenesis of glioma was further elucidated. Finally, Q11 as an effective glioma anti‐inflammatory agent was evaluated, with the goal to provide a perspective on anti‐inflammatory GBM therapy and offer a new potential treatment strategy for GBM.

## 2 Experimental Section

### 2.1 Clinical Tissue Samples

A total of 34 paraffin tissue samples of glioma were obtained from the Pathology Department of the Second Affiliated Hospital of Zhengzhou University from June 2014 to May 2019. Inclusion criteria included: 1) Confirmed pathologically as glioma after surgical resection, including WHO grade I to IV; 2) 18–70 years old; 3) no chemotherapy or radiotherapy was received before surgery; 4) no history of other tumors or systemic chronic diseases (such as hepatitis, tuberculosis, diabetes, etc.). Glioma samples included 1 ganglioglioma (Grade I), two angiocentric gliomas (Grade I), and 10 oligodendrogliomas and astrocytomas (Grade II), five anaplastic astrocytoma (Grade III), and 13 GBM (Grade IV) according to the 2021 WHO classification criteria for CNS tumors. Peritumoral tissue in paraffin specimens was defined as normal brain tissue as evaluated under light microscopy at a high power field (400×) away from the tumor area. Survival time was defined as starting from the day of surgery (day 1) to the date of last follow‐up or loss of follow‐up; if the patient died before the follow‐up period, the end time was the date of death. Thirty‐two normal brain tissues were collected from the Forensic Center of Zhengzhou University. The inclusion criteria were as follows: 1) Brain specimens were fixed within 12 h after death; 2) 20–70 years old; 3) no history of cancer or chronic diseases (such as hepatitis, tuberculosis, diabetes, etc.). All paraffin brain samples were used for tissue microarray analysis. Of the 34 glioma patients, 31 were tissue intact after tissue microarray.

Twelve freshly frozen glioma and adjacent tissue samples were collected from the Neurosurgery Department of Henan Tumor Hospital from March 1, 2020 to November 31, 2021. All patient tissues were confirmed pathologically as gliomas, including 4 astrocytoma and 8 GBM. For each sample, tumor and peritumoral tissue was processed separately. Peritumoral tissue was defined as cortical tissue in nonfunctional areas more than 1 cm away from the surgical margin of the tumor. Among the six normal brain specimens, three were cerebral cortex tissues of non‐functional areas of patients with cerebral trauma, which were collected from the First Affiliated Hospital of Zhengzhou University, and three were normal brain tissues of cerebral hemangioma, which were collected from Henan Tumor Hospital. Tissue samples were transported to the laboratory immediately from the operating room to begin dissociating samples within 1 h of resection.^[^ ^40^ ^]^ This study was approved by the Bioethics Review Committee of Zhengzhou University (ZZUIRB2022‐152), and informed consent was obtained from all the specimen donors or their families.

### 2.2 Design and Screening of the CYP2E1 Inhibitor

Based on the crystal structure of CYP2E1, drug design software of Schrodinger and SYBYL,^[^ ^41^, ^42^ ^]^ and compound libraries of Leads Now and Specs were used for computer‐aided drug design and virtual screening of potential CYP2E1 inhibitors. Some compounds with reasonable binding patterns and high scores were selected based on computer aided drug design. Based on inhibition and selectivity in vitro and in vivo, a comparison of Q11 and screened small molecule compounds found that Q11 was the most dominant lead compound, followed by a series of structural optimization and function screening for Q11 carried out.

#### 2.2.1 Synthesis of Q11 Series Compounds

Compound 1 (1.0 equiv) was dissolved in 2 m NaOH (1.1 equiv) aqueous solution for 0.5 h monitored by TLC. Then, the pH of reaction system was adjusted to 2–3 using concentrated hydrochloric acid to precipitate white solid, filter solid and dry to constant weight under vacuum to afford compound 2. To a stirring system of compound 2 (1.0 equiv) dissolved in DCM, CDI (1.2 equiv) was added in batch to activate compound 2 for 1 h. After that, N,O‐Dimethylhydroxylamine hydrochloride (1.2 equiv) and Et3N (2.0 equiv) were added into system. Reaction was over after 0.5 h monitored by TLC. Then, 20% HCl aqueous solution was added to adjust pH to 2–3, system was extracted with DCM for 3 times, organic layer was collected, washed with water, and evaporated to obtain compound 3. Under the nitrogen atmosphere, compound 3 was dissolved in anhydrous tetrahydrofuran and cooled to −20 °C, methyl magnesium chloride was slowly added. Reaction was over after 1 h monitored by TLC. Then, system was quenched using saturated salt water, and extracted with ethyl acetate. The combined organic phase was dried by anhydrous Na_2_SO_4_ and evaporated to obtain Q11, then purified by vacuum distillation.

Reagtions and conditions (a) NaOH, H_2_O, 1 h; (b) CDI, N,O‐Dimethylhydroxylamine hydrochloride, Et_3_N, DCM, 1.5 h; (c) CH_3_MgCl, dry‐THF, −20 °C

#### 2.2.2 In Vitro Inhibition

Chlorzoxazone with a concentration of 62.5 µm and inhibitors with different gradient concentrations were selected to plot the inhibition of chlorzoxazone and to calculate the IC_50_ of the inhibitor. A Lineweaver–Burk curve was used to determine the mechanism and calculate the *K*_i_ of inhibitors of CYP2E1.

#### 2.2.3 Surface Plasma Resonance

Surface Plasma Resonance (SPR) experiments^[^ ^43^ ^]^ were carried out using the Biacore system (Biacore 8k, Cytiva). Recombinant CYP2E1 protein was purified by Cloud‐Clone Corp. Wuhan. Running buffer contained PBS (2 mm KH_2_PO_4_, 10 mm Na_2_HPO_4_, 137 mm NaCl, 2.7 mm KCl) with 0.005% Tween‐20, pH 7.4. CYP2E1 protein was diluted to 25 µg mL^−1^ in immobilization buffer and bound to a CM5 chip at about 2000RU. Immobilization buffer contained 10 mm sodium acetate, pH 4.0. Samples of compound Q11 were diluted in running buffer with 125, 62.5, 31.25, 15.625, 7.8, 3.9, 1.95 µm and injected at 30 µL min^−1^ for 60 s contact time followed by dissociation for 120 s. The data was fitted with Biacore Insight Evaluation software (Version 2.0).

#### 2.2.4 Selectivity of Q11

A 100 µL microsomal incubation system^[^ ^44^ ^]^ contained probe drugs for each CYP with different series of concentrations, Q11, phosphate buffer, EDTA, magnesium chloride, and substrates (phenacetin for CYP1A2, coumarin for CYP2A6, bupropion for CYP2B6, paclitaxel for CYP2C8, tolbutamide for CYP2C9, omeprazole for CYP2C19, dextromethorphan for CYP2D6, chlorzoxazone for CYP2E1, midazolam for CYP3A4/5). HPLC‐UV and HPLC‐FLD were used to measure the number of metabolites.

#### 2.2.5 In Vivo Inhibition of Q11 in Rats

A self‐controlled cross‐administration experiment was designed based on previous report.^[^ ^45^ ^]^ In the first round of the experiment, a single intraperitoneal injection of diethylnitrosamine at 50 mg kg^−1^ was given in the first week. After a 1‐week interval, the second round of the experiment was conducted, where each rat was given Q11 at 6, 30, or 150 mg kg^−1^ (i.g), and then, after 5 min, an intraperitoneal injection of diethylnitrosamine at 50 mg kg^−1^ was given. Collection of orbital blood was carried out at 2, 7, 15, 30 min and 1, 2, 4, 6, 9, 12, 24, 36, 48, 60 h.

### 2.3 Bioavailability and Pharmacokinetics of the CYP2E1 Inhibitor Q11

#### 2.3.1 Bioavailability of Q11 in Rats

Eight healthy male SD rats were randomly divided into 2 groups (4 rats in each group) using a double‐period self‐cross control principle. The first group was given Q11 (30 mg kg^−1^, i.g), and the second group was given Q11 (30 mg kg^−1^, i.v.). One week later, a second round of experiments was conducted in which Q11 (30 mg kg^−1^, i.v.) was given to the first group of rats and Q11 (30 mg kg^−1^, i.g) was given to the second group. Collection of orbital blood was carried out at 0, 2, 7, 15, 30 min and 1, 2, 4, 6, 9, 12, 24 h after Q11 administration.

#### 2.3.2 Pharmacokinetics of Q11 in Rats

Thirty healthy male SD rats were randomly divided into three equal groups. Q11 was administrated at 6, 30, or 150 mg kg^−1^ (i.g) to these three groups. Collection of orbital blood was carried out at 0, 2, 7, 15, 30 min and 1, 2, 4, 6, 9, 12, 24, 36 h after Q11 administration.

### 2.4 Immunohistochemistry and Immunofluorescence

Paraffin‐embedded specimens were cut into 4‐µm sections. After deparaffinization with xylene and rehydration, antigen retrieval was performed. For immunohistochemistry (IHC), endogenous peroxidase activity was blocked with 3% H_2_O_2_ in methanol. Primary antibodies against CYP2E1 (1:200, Abcam, ab28146), Iba‐1 (1:150, Servicebio, China), CD206 (1:150, Servicebio), and CD86 (1:150, Servicebio) were used and results were evaluated blindly by two pathologists and quantified by IPP 6.0 soft. Immunofluorescence staining was performed as described previously.^[^ ^46^ ^]^

### 2.5 Western Blot

Tissues and cells were harvested and lysed with RIPA buffer. The protein concentration was determined using a BCA kit. Samples were separated on 10% SDS‐PAGE gels and blotted onto nitrocellulose membranes (Millipore).The membranes were incubated with primary antibodies at 4 °C overnight, washed three times with TBST, and then incubated with HRP‐conjugated anti‐mouse IgG or anti‐rabbit IgG diluted in TBST containing 1% non‐fat milk at room temperature for 1 h. After final washing with TBST, the membranes were developed by using ECL.

### 2.6 Quantitative Real‐Time PCR

Total RNA was isolated from cells using Trizol (Thermo Fisher Scientific). For qRT‐PCR analyses of mRNAs, first‐strand cDNA was synthesized using a PrimeScript qRT‐PCR kit (Takara). The expression levels of target genes were determined with specific primers. The primers are listed in Table S1, Supporting Information.

### 2.7 Measurement of Rat CYP2E1 Activity

CYP2E1 activity was evaluated by the pharmacokinetic characteristics of chlorzoxazone (CZX), a probe substrate of CYP2E1. Seventeen female SD rats (6–8 weeks, 165 ± 15 g) were given a CZX solution orally at a dose of 15 mg kg^−1^ (i.v.) after an overnight fast of ≥12 h (with water allowed ad libitum). A total of 300 µL blood was collected from orbit at 0, 5, 15, 30, 60, 90, 120, and 180 min before and after administration and placed in an anticoagulant tube containing heparin. The plasma was separated and the concentration of CZX in plasma was determined by high performance liquid chromatography (HPLC). Pharmacokinetic parameters (*t*_1/2_, AUC, CL, etc.) obtained by DAS software were used to characterize the activity of CYP2E1 in rats.

### 2.8 Cell Experiments

Glioma cell lines (GL261, C6, and U251) and microglia/macrophage (M/M*φ*) cell lines (RAW264.7, BV‐2, THP‐1, and HMC‐3) were obtained from the American Type Cell Culture Collection (ATCC). GL261, C6, U251, RAW264.7, and BV‐2 cells were cultured in Dulbecco's modified Eagle's medium (DMEM, Gibco) supplemented with 10% fetal bovine serum (FBS, Gibco). THP‐1 and HMC‐3 cells were cultured in RPMI 1640 medium (Gibco) and MEM medium (Gibco), respectively. Cells were incubated at 37 °C with 5% CO_2_. Wild type and *Cyp2e1* knockout (KO) primary microglial were obtained from brains of newborn C57BL/6 mice (0–48 h) by modified methods based on a previous report.^[^ ^47^, ^48^ ^]^ Briefly, brain cortices were mechanically dissociated and suspended in DMEM/F12 with Glutamic I (Gibco, CA, USA) supplemented with 10% FBS and 1% v/v penicillin‐streptomycin. Cells obtained from newborn mice brains were seeded at a T75 culture fast and incubated at 37 °C in 5% CO_2_, with half medium replacement every 3 days. Microglia were shaken at 200 rpm for 4 h after 10 days of culture. The isolated primary microglia were seeded in 6‐well plates or 24‐well plates at a desired density (1.4 × 10^6^ and 5.0 × 10^5^ cells per well, respectively). For the induction of the KO group, primary microglia were induced by LPS (100 ng mL^−1^) for 6 h. For co‐culture, M/M*φ* and primary microglia were induced by IL‐4 (10 ng mL^−1^) and IL‐13 (10 ng mL^−1^) and treated with or without different concentrations of Q11; after incubation for 24 h, the cell supernatant was used as conditioned medium for indirect co‐culture with glioma cells. Plasmid transfection in HMC‐3 was performed according to commercial kit instructions by using Lipofectamine 3000 (Thermo Fisher). Proliferation, apoptosis, and cell cycle of glioma cells were detected by CCK–8 kit, Annexin V‐FITC/PI staining, and cell cycle kit (KeyGEN bioTECH, Jiangsu, China), respectively, according to the manufacturer's instructions. Migration was performed by wound assays, taking 24 h as the final time point.

### 2.9 Transcriptomic Sequencing

Transcriptome sequencing was performed in collaboration with Wekemo Tech Group Co., Ltd. Shenzhen China.

### 2.10 In Vivo Animal Experiment and Treatments

Mice and rats with a *Cyp2e1* knock out (*Cyp2e1^−/−^*) were generated by Beijing Biocytogen Co., Ltd. Co‐housed *Cyp2e1* wild‐type littermate mice and rats (*Cyp2e1^+/+^*) were used as control animals in the target discovery and validation part of the experiments. Female C57BL6J mouse (7–8 weeks), SD rats (180–200 g), and BABL/C nude mice (7–8 weeks) were purchased from Beijing Vital River Laboratory Animal Technology Co., Ltd. (Beijing, China). For the mouse models, GL261 (1 × 10^6^ cells) or U251‐LUC (5 × 10^6^ cells) in 5 µL of DMEM was injected intracranially into the C57BL6J mice or BABL/C nude mice by stereotactic surgery (2 mm left and 1 mm anterior to the bregma, 3 mm deep from the dura). For the rat model, C6 (1 × 10^5^ cells) in 6 µL of DMEM were injected intracranially into the S–D rats by stereotactic surgery (2 mm left and 1 mm before the bregma, 3.7 mm deep from the dura). Q11 was administered 3 days before the transplanted tumor model, and TMZ was administered 3 days after the operation. The experiment was terminated when the mice in the model group become moribund, and all death dates were recorded. Tumor volumes were calculated using the formula volume = 0.5 × length × width.^[^ ^2^ ^]^ All experimental mice were bred and maintained under specific pathogen‐free (SPF) conditions, fed standard laboratory chow, and kept on a 12 h light/dark cycle and temperature and humidity were kept at 22 ± 2 °C, 55% ± 5%. The survival of mice was observed and analyzed using the Kaplan–Meier method. Bioluminescence imaging was performed on day 14th after transplantation by a blinded investigator to monitor tumor growth in vivo using an IVIS imaging system (Xenogen), and the intensities of bioluminescence signals were compared statistically between the groups. For the pharmacokinetics study, SD female rats aged 6–8 weeks were administered Q11 (30 mg kg^−1^) by intragastric administration. Afterward, plasma, normal brain cortex, and hippocampus tissues were harvested at different times (0, 0.5, 1, and 4 h after intragastric administration). Brain tissue and plasma were collected from each mouse at each time point and quickly frozen for future analysis. The concentration of Q11 was measured by high performance liquid chromatography (HPLC, Agilent). All animal experiments were approved by the Animal Experiment Administration Committee of Zhengzhou University (ZZUIRB2022‐152).

### 2.11 Statistical Analysis

Data were represented as the as mean ± SD or mean ± SEM of a minimum of three biological replicates. Statistical analysis was processed with SPSS 23.0. For normal measurement data, a *t*‐test was used for comparison between two groups and one‐way ANOVA was used for pairwise comparison between multiple groups. Non‐normal data was tested using the non‐parametric Mann–Whitney U test.

## 3 Results

### 3.1 Discovery and Validation of CYP2E1 as a Potential Target of Glioma

**Figure 1:**
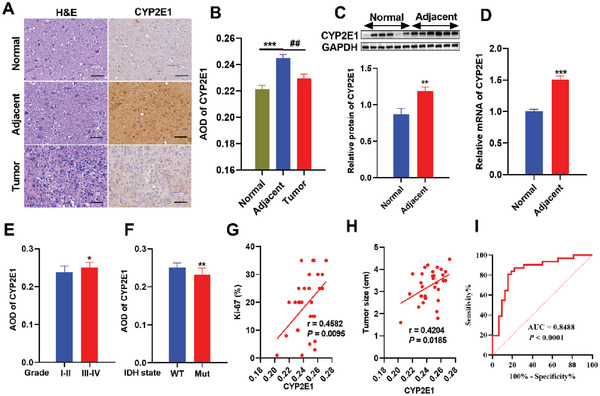
Up‐regulated expression of CYP2E1 in the tumor microenvironment of glioma patients and its clinical significance. A) Representative immunohistochemical images of CYP2E1 expression in normal brain, para‐tumor brain, and tumor tissue. B) Immunohistochemical quantification. C) Western blot bands and quantitative results of CYP2E1 in normal brain and para‐tumor brain tissues. D) mRNA levels of CYP2E1 in normal brain and para‐tumor brain tissues. E) Expression of CYP2E1 in the high‐grade group was higher than that in the low‐grade group. F) Expression of CYP2E1 in the wild‐type group was higher than that in the mutant group. Expression of CYP2E1 was positively correlated with G) tumor size and H) Ki67 proliferation index. I) The receiver operator characteristic curve (ROC) of CYP2E1 content for predicting glioma occurrence. (A, B) Normal, *n* = 32; Adjacent and Tumor, *n* = 31. (C, D) Normal, *n* = 6; Adjacent, *n* = 12. (E–I, II) *n* = 6; (E–III, IV) *n* = 12. (F) WT, *n* = 12; Mut, *n* = 19. (G, H) *n* = 31.

Immunohistochemical staining on tissue microarray showed that CYP2E1 was significantly elevated in brain tissues adjacent to glioma both in protein (**Figure** **1**A–C) and mRNA levels (Figure 1D). In addition, CYP2E1 in the high‐grade (WHO grade III‐IV) group was significantly higher than that in low‐grade (WHO Grade I‐II) group (Figure 1E), and the IDH1/2 mutant group was significantly lower than that in IDH1/2 wild‐type group (Figure 1F). In addition, CYP2E1 was positively correlated with tumor size (Figure 1G, *r* = 0.4204, *p* = 0.0185) and tumor proliferation index (Figure 1H, *r* = 0.4582, *p* = 0.0095), indicating that CYP2E1 in TME is related to the higher malignancy of glioma. The receiver operator characteristic curve (ROC) plotted by CYP2E1 content in normal brain tissue and adjacent tissue of glioma patients was used to predict the occurrence of glioma, which showed that the area under ROC curve (AUC) was 0.849 (95%CI, 0.768–0.941, *p* < 0.001), with a sensitivity of 87.1% and a specificity of 78.3% (Figure 1I). These results suggest that CYP2E1 of TME may be related to the occurrence and progression of glioma.

### 3.2 Validation of CYP2E1 as a Potential Target of Glioma

**Figure 2:**
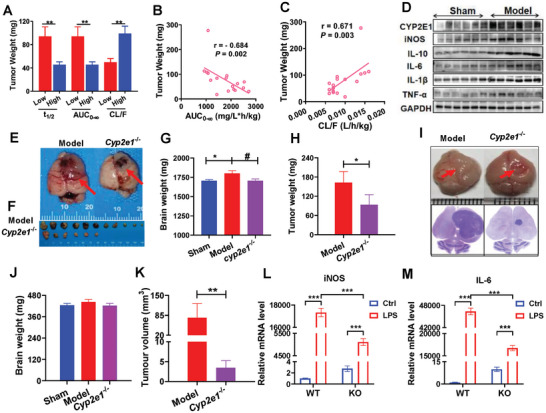
Validation CYP2E1 as a potential target of glioma. A–D) The causal relationship between the chlorzoxazone (CZX) metabolism by CYP2E1 and the severity of glioma model in rats. A) Significant differences in glioma tumor weight varied by pharmacokinetic parameters of chlorzoxazone after i.p. administration (50 mg kg^−1^) metabolized by CYP2E1 in female Sprague–Dawley (S–D) rats. Correlation between B) AUC0‐∞ (mg/L*h), C) CL/F (L h^−1^ kg^−1^) of CZX and glioma tumor weight. D) Change of CYP2E1 and inflammation in C57 mouse glioma model inoculated with GL261 cells in situ. E–G) *Cyp2e1^−/−^* suppressed the growth of C6 orthotopic tumors in SD rats. E) Pictures of the whole brain with the tumors from various groups of the C6 orthotopic model; the red arrow indicates the tumor. F) Images of tumor. G) Brain weight. H) Tumor weight. I–K) *Cyp2e1^−/−^* suppressed the growth of GL261 orthotopic tumors in mice. I) Pictures of the whole brain and H&E staining with the tumors from various groups of the GL261 orthotopic model, the red arrow indicates the tumor. Scale bar = 50 µm. J) Brain weight. K) Tumor volumes. L,M) Effect of *Cyp2e1^−/−^* on mRNA levels of inflammation cytokines in primary microglia induced by LPS. L) iNOS. M) IL‐6. (A) Low, *n* = 9; High, *n* = 8. (B, C) *n* = 17. (D) Sham, *n* = 6; Model, *n* = 6. (F–H), Model, *n* = 12; *Cyp2e1^−/−^*, *n* = 6. (I, J) Model, *n* = 10; *Cyp2e1^−/−^*, *n* = 6.

To further clarify whether CYP2E1 is involved in the tumorigenesis of glioma, we evaluated the relationship between CYP2E1 activity and glioma in rats. GBM‐bearing rats were divided into two groups according to the pharmacokinetic parameters *t*_1/2_ or AUC_0‐∞_ or clearance (CL) of CZX in rats. The tumor weight of the high *t*_1/2_ and AUC_0‐∞_ groups was significantly lower than that of the low *t*_1/2_ and AUC_0‐∞_ groups. And for CL, the trend was reversed. Further analysis indicated positive correlation between CYP2E1 activity and tumor severity in GBM rats, evidenced by significantly inverse correlation for AUC_0‐∞_ (**Figure** **2**B) and positive correlation for CL (Figure 2C) with tumor weight. Moreover, a mouse GBM model showed significantly increased CYP2E1 expression, accompanied by significantly increased inflammation. These results show that increased activity or content of CYP2E1 can promote the development of glioma, which may be related to inflammation.

To test CYP2E1 as therapeutic target, we next established *Cyp2e1* knockout (*Cyp2e1^−/−^*) mice and rats. As expected, tumors in the *Cyp2e1^−/−^* group were significantly smaller than those in the wild‐type group in glioma‐bearing SD rats (Figure 2E–G). Knockout of *Cyp2e1* significantly attenuated brain weight and tumor weight in intracranial GBM xenograft rats, in agreement with those in *Cyp2e1* knockout mice (Figure 2H–J), results suggesting a prompting role of CYP2E1 in glioma. Furthermore, *Cyp2e1^−/−^* exhibited reduced levels of inflammation on in primary microglia induced by lipopolysaccharide (LPS) (Figure 2K,L). In addition, *Cyp2e1* knockout significantly improved the general state of glioma‐bearing rats and counteract the tumor‐induced weight loss (Figure S1A,B, Supporting Information). These results suggested that CYP2E1 is an attractive potential target for glioma.

**Figure 3:**
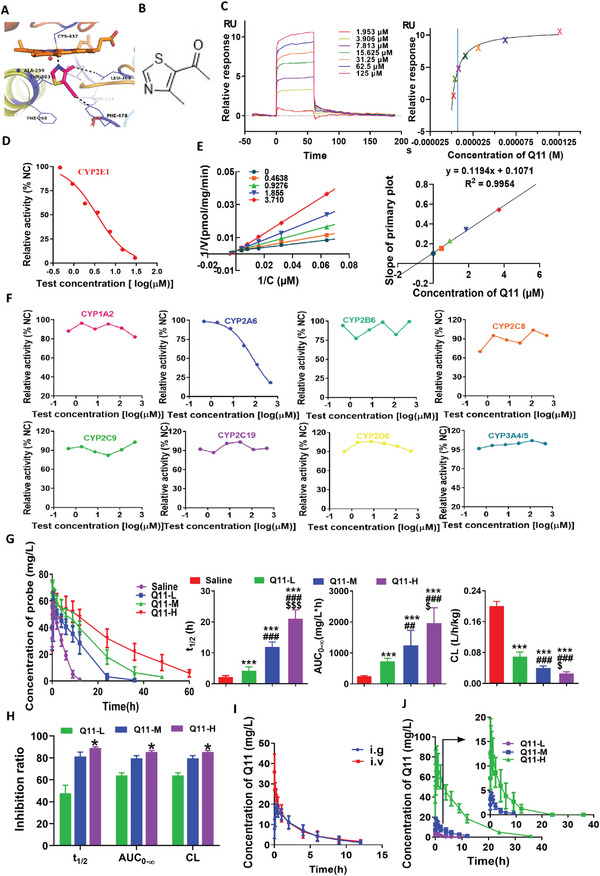
Research and development of a specific CYP2E1 inhibitor, Q11. A) Molecular docking of Q11 into the target site of CYP2E1. B) Chemical structure of 1‐(4‐methyl‐5‐thialzolyl) ethanone, defined as Q11. C) Determination of *K*_D_ for binding between Q11 and CYP2E1 using surface plasmon resonance. D) Half‐inhibitory concentration (IC50) of inhibition of Q11 on CYP2E1. E) Lineweaver–Burk plots and a secondary plot of the inhibition by Q11 on CYP2E1. F) Selectivity of Q11 on inhibition of the remaining nine CYPs. G,H) In vivo inhibition by Q11 of CYP2E1 activity determined by metabolism of diethylnitrosamine (DEN) in rats, saline (*n* = 27), Q11‐L (*n* = 10), Q11‐M (*n* = 9), and Q11‐H (*n* = 8). I,J) Bioavailability (*n* = 8) and pharmacokinetics (*n* = 10) of Q11. Q11‐L (6 mg kg^−1^), Q11‐M (30 mg kg^−1^), and Q11‐H (150 mg kg^−1^). Data are expressed as the mean ± S.D.

To analyze target security, *Cyp2e1^−/−^* rats were selected for growth and developmental observation for 36 months. The results showed no obvious pathological changes in blood biochemical indexes (**Figure** **3**; Figure S2, Supporting Information) and organs (Figure S4, Supporting Information) compared with control littermates, suggesting that CYP2E1 is a safe potential target for glioma.

### 3.3 Research and Development of a Specific CYP2E1 Inhibitor

To screen of a CYP2E1 inhibitor, traditional virtual screening from compound libraries were conducted basing on the crystal structure of CYP2E1, which led to more than 100 potential compounds with a reasonable binding mode with CYP2E1 with low binding energy and high prediction score; however, none showed strong inhibition with high selectivity of CYP2E1 by in vitro inhibition experiments. Chlormethiazole, an inhibitor of CYP2E1, is metabolized into Q11, 1‐(4‐methyl‐5‐thialzolyl) ethanone in vivo. Therefore, we tested the activity of Q11 toward CYP2E1. The binding of Q11 to CYP2E1 was determined using SPR which yielded a KD of 7.0 µm (Figure 3C) and Q11 was an inhibitor of CYP2E1 with a IC50 of 1.64 µm (Figure 3D).

To evaluate the structure–activity relationship of Q11, a similarity search using Q11 as the query structure in combination with a subsequent molecular docking study led to the identification of compounds (Table S2, Supporting Information). Bioelectronic isostere replacement of the thiazole group to pyridine, pyrrole, indole, oxazole, led to compounds. To study the structural‐activity relationship of the acetyl group, we synthesized compounds in which the acetyl group was replaced with a methyl group, imine group, or *α*,*β*‐unsaturated ketone compounds. In addition, we also tried to replace the methyl group on the thiazole ring with ethyl and replace the hydrogen on the thiazole ring with a methyl or amino group. However, activity tests showed that these compounds had very weak activity toward CYP2E1. Thus, we selected Q11 as the lead compound.

Further in vitro inhibition of CYP2E1 indicated that Q11 acted as a competitive‐noncompetitive mixed‐type inhibitor with a *K*_i_ of 0.897 µm (Figure 3E). Only slight inhibition of CYP2A6 was found with an IC_50_ of 76.2 µm, which was a high concentration difficult to achieve in the body, and no inhibition of other CYP enzymes was found (Figure 3F), suggesting strong selectivity for CYP2E1. In vivo inhibition demonstrated inhibition ratio of 85.42%, 79.63% and 63.98% for Q11 at a dose of 150, 30, and 6 mg kg^−1^ when evaluated in terms of clearance (CL) on the metabolism of diethylnitrosamine (DEN) in a self‐controlled cross‐administration experiment (Figure 3G,H). These results indicate that Q11 is a potent and selective CYP2E1 inhibitor.

Comparative pharmacokinetics of Q11 in rats after oral and intravenous administration at 30 mg kg^−1^ yielded an average bioavailability at 94.65% (Figure 3I), indicating excellent absorption after oral intake. Following oral administration Q11 was rapidly absorbed with a mean *T*_max_ at 0.45 h, and a mean *t*_1/2_ at 3.05 h. Additional pharmacokinetic parameters of Q11 showed proportional changes in rats with a dose ranging from 6 to 150 mg kg^−1^ (Figure 3J).

### 3.4 Anti‐Glioma Effect of the CYP2E1 Inhibitor Q11 In Vivo

**Figure 4:**
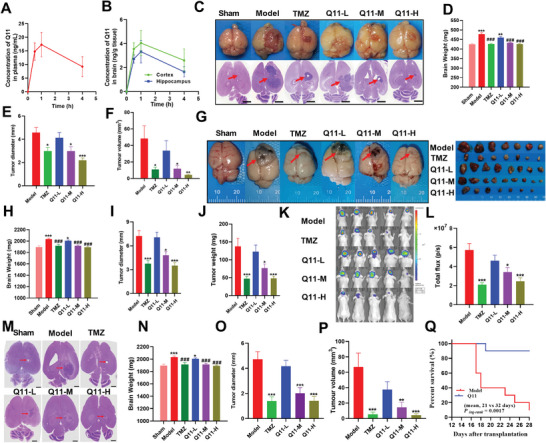
Inhibition of CYP2E1 by Q11 decreased GBM growth and prolonged the survival time in vivo. A,B) In vivo biodistribution analysis of Q11. C–F) Q11 inhibited the growth of glioma in an C57BL/6 mouse intracranial orthotopic model induced by GL261 cells. C) Representative general images and HE pictures. D) Brain weight. E) Tumor diameter. F) Tumor volumes. G–J) Q11 inhibited the growth of glioma in S–D rats’ intracranial orthotopic model induced by C6 cells. G) Representative general images of brain and tumor. H) Brain weight. I) Tumor diameter. J) Tumor weight. K–P) Q11 inhibited the growth of glioma in a BABL/C nude mouse intracranial orthotopic model induced by U251‐LUC cells. K) Representative bioluminescence imaging of animals at 14 days after intracerebral implantation of luciferase‐transfected U251 cells. L) Quantification of fluorescence (p s^−1^ cm^−2^ sr^−1^) data for U251‐LUC glioma‐bearing mice. M) Representative HE pictures of mice. N) Brain weight. O) Tumor diameter. P) Tumor volumes. Q) Kaplan–Meier survival curves of mice with intracerebral glioma treated with saline or Q11 (30 mg kg^−1^). (A, B), *n* = 6. (C–F) Sham, *n* = 10; Model, *n* = 26; TMZ, *n* = 26; Q11‐L, Q11‐M, and Q11‐H, *n* = 25. (G–J) Sham, *n* = 10; Model, *n* = 22; TMZ, *n* = 21; Q11‐L, *n* = 20; Q11‐M and Q11‐H, *n* = 21. (K–P) Sham, *n* = 10; Model, TMZ, Q11‐L, Q11‐M, and Q11‐H, *n* = 15. (Q) *n* = 20.

To test the blood brain barrier (BBB) permeability of Q11, Q11 (30 mg kg^−1^) was given by intragastric administration and plasma, brain cortex, and hippocampus tissues were harvested. Surprisingly, the plasma concentration of Q11 reached a peak at about 1 h and gradually decreased from 14.75 to 9.21 ng mL^−1^ at 4 h after intragastric administration (**Figure** **4**A). Q11 in cerebral cortex and hippocampus tissues changed in parallel with plasma concentration (Figure 4B), suggesting Q11 is capable of penetrating BBB to achieve a functionally relevant therapeutic concentration.

To evaluate the anti‐glioma effect of Q11, three orthotopic glioma models were established. In the C57BL/6 mouse intracranial orthotopic model induced by GL261 cells, Q11 significantly inhibited the growth of glioma (Figure 4C), evidenced by brain weight (Figure 4D), tumor diameter (Figure 4E), and volumes (Figure 4F). These findings were consistent with those of the Sprague–Dawley (SD) rat intracranial orthotopic model induced by C6 cells. Oral administration of Q11 (30 mg kg^−1^) significantly suppressed the growth of tumor (Figure 4G), evident by brain weight (Figure 4H), tumor diameter (Figure 4I), and tumor weight (Figure 4J). In nude model mice induced by luciferase‐labelled U251 cells, the intracranial tumor was monitored by bioluminescence using a Xenogen IVIS system. Bioluminescence imaging showed that lesser bioluminescence accumulated in tumor sites of mice in the TMZ group, Q11‐M (Middle, 10 mg kg^−1^) and Q11‐H (High, 30 mg kg^−1^) groups on day 14 after transplantation (Figure 4K,L). Endpoint tumors on day 36 after transplantation were collected and measured, which showed that the orthotopic tumors in the model group progressed aggressively (Figure 4M), while TMZ and Q11 treatment markedly suppressed brain weight (Figure 4N), as well as tumor diameter (Figure 4O) and tumor volume (Figure 4P). In addition, Q11 administration significantly prolonged the survival time of mice with intracerebral tumors (mean, 32 vs 21, *P*_log‐rank_ = 0.0017, Figure 4P). Collectively, these results indicate that Q11 significantly inhibits the growth of glioma and prolongs survival time of glioma‐bearing mice, with an inhibitory effect superior to that of TMZ.

### 3.5 Anti‐Glioma Effect of the CYP2E1 Inhibitor Q11 In Vitro

**Figure 5:**
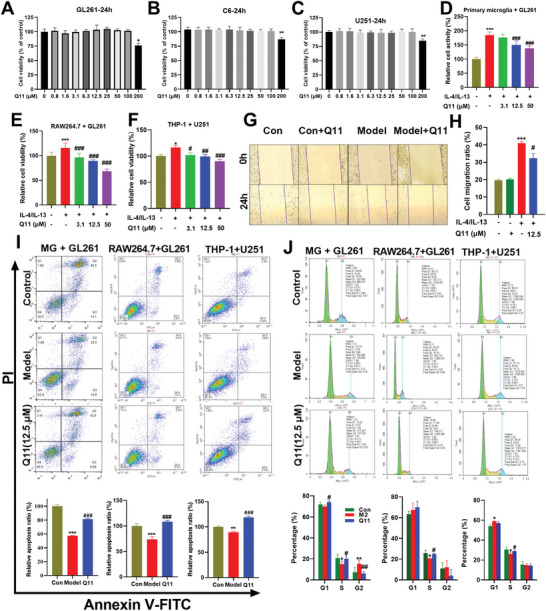
Inhibition of CYP2E1 by Q11 in vitro inhibited the proliferation and migration of glioma cells, promoted apoptosis, and arrested the cell cycle in S phase. A–C) Q11 has no direct inhibitory effect on GL261, C6, and U251 glioblastoma cells. D–F) Inhibition of CYP2E1 by Q11 inhibited the proliferation of GL261, C6, and U251 cells when co‐cultured with microglia/macrophages. G,H) Inhibition of CYP2E1 by Q11 inhibited the migration of GL261 cells, when co‐cultured with microglia/macrophages. I) Inhibition of CYP2E1 by Q11 accelerated GL261, C6, and U251 cells apoptosis when co‐cultured with microglia/macrophages. J) Inhibition of CYP2E1 by Q11 induced S‐phase arrest in GL261 cells when co‐cultured with primary microglia.

To explore the anti‐tumor mechanism of Q11, we used different concentrations of Q11 in incubations with tumor cells. To our surprise, Q11 did not directly kill GL261 cells (**Figure** **5**A), C6 (Figure 5B), or U251 cells (Figure 5C) at concentrations below 100 µm, but significantly inhibited the proliferation of GL261 or U251 cells when co‐cultured with primary microglia cells (Figure 5D), RAW 264.7 (Figure 5E), or THP‐1 macrophages (Figure 5F). Likewise, Q11 restrained migration (Figure 5G,H) and promoted apoptosis (Figure 5I) of GL261 cells, as well as arrested the cell cycle in S phase (Figure 5J). Together, these results suggest that Q11 inhibits glioma growth through M/M*φ* of TME.

**Figure 6:**
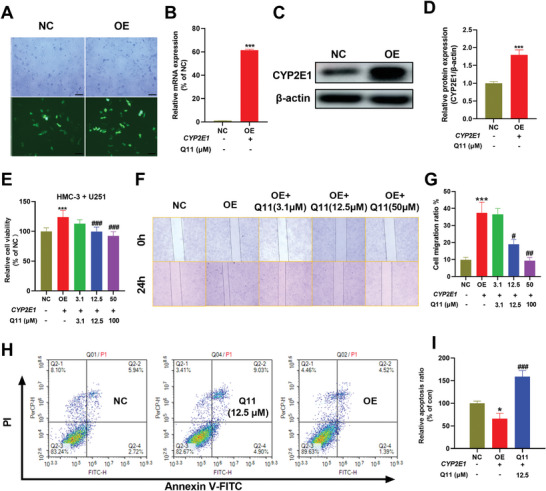
Upregulated CYP2E1 in HMC‐3 cells promoted proliferation and migration and inhibited apoptosis of U251 glioma cells. A) Representative morphology and fluorescence images of HMC‐3 cells after transfection with CYP2E1 under an inverted microscope. Scale bar = 200 µm. B) CYP2E1 mRNA level in NC and OE groups was measured by qPCR. C) Western blot protein bands of CYP2E1. D) Quantitative of WB protein bands. E) Overexpression of CYP2E1 in HMC‐3 cells promoted the proliferation of U251 glioma cells. F,G) Inhibition of CYP2E1 by Q11 inhibited the migration of GL261 cells. H,I) Overexpression of CYP2E1 in HMC‐3 cells suppressed U251 tumor cell apoptosis. Data are presented as the mean ± SD. *p < 0.05, **p < 0.01, ***p < 0.001 versus NC, ^##^ *p* < 0.05, ^##^ *p* < 0.01, ^###^ *p* < 0.001 versus OE group. CM, conditioned medium. NC, negative control. OE, overexpression.

To investigate the role of CYP2E1 in glioma TME, we transfected CYP2E1 plasmids into HMC3 microglia and overexpression efficiency was verified by a fluorescence signal under an inverted microscope (**Figure** **6**A), RT‐qPCR (Figure 6B), and Western blotting (Figure 6C,D). Upregulation of CYP2E1 in HMC‐3 significantly promoted proliferation (Figure 6E) and migration (Figure 6F,G), and inhibited apoptosis (Figure 6H,I) of U251 cells, suggesting CYP2E1 is involved in tumorigenesis or progression of glioma. In addition, the tumor‐promoting effects of upregulating CYP2E1 in HMC‐3 were reversed by Q11 (Figure 6E–I).

### 3.6 Q11 Inhibited Growth of Glioma by Regulating Microglia/Macrophage Reprogramming

**Figure 7:**
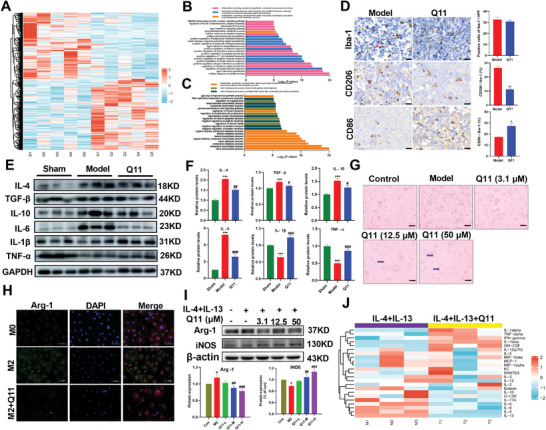
Effect of Q11 on the tumor microenvironment of glioblastoma‐bearing mice. A) Heat map of differentially‐expressed genes between the model group and the Q11 group, *n* = 5 for each group. B) Up‐regulated genes and C) down‐regulated genes in the Q11 group in biological processes. D) Representative staining images of Iba‐1, CD206 and CD86 staining on tumor tissues. E,F) Effects of Q11 on inflammatory factors in the tumor microenvironment of a C6 glioma cell orthotopic xenograft model in rats. G) Representative microscopic view of primary microglia in high‐power (200×) fields. The red arrow shows a typical amoeba‐like M2 microglia with fewer stubby projections and roundish cells. The blue arrow shows “branching” or “multiple protuberant” M1‐like microglia. Scale bar, 100 µm. H) Immunofluorescence staining of Arg‐1 (green) and DAPI (blue) in primary microglia. Scale bar = 50 µm. I) Expression of Arg‐1 and iNOS evaluated by a western blot analysis. J) Heat map of differential expression of cytokines at the protein level in cell culture medium of primary microglia with or without Q11 treatment. For (D–F), *n* = 6 for each group.

RNA‐Seq was performed to investigate effect of Q11 on TME. A total of 595 differentially expressed genes (DEGs) were identified according to *p* ≤ 0.05 between the glioma model group and the Q11 treatment group (**Figure** **7**A), including 308 up‐regulated genes and 287 down‐regulated genes. Gene ontology enrichment analysis found that up‐regulated biological processes were significantly related to inflammation and immunity, including biosynthesis, metabolism and secretion of cytokines, activation of immune cells and their mediated immune responses, and signal transduction (Figure 7B), while down‐regulated genes revealed enrichment in biosynthesis and metabolism of lipids, steroids, sterols, and cholesterol (Figure 7C). Morphology of Iba‐1 (microglia marker)‐positive cells in the model group was characterized by a retraction and thickening of processes, showing a typical ameboid shape, with the morphological characteristics of M2 microglia (Figure 7D). Conversely, Q11 lead to the morphological transition of microglia from the ameboid to the inflammatory protrusions state, showing typical “multi‐protrusion” or “branching” cells, with the morphological characteristics of M1‐type microglia. CD206 (M2 marker) and CD86 (M1 marker) staining showed that the number of CD206 positive cells in Q11 group was significantly reduced, and in contrast, CD86‐positive cells increased significantly. In addition, M2‐like inflammatory factors, such as IL‐4, TGF‐*β*, IL‐10, VEGFB, and IL‐6 were significantly increased, while M1‐like inflammatory factors (e.g., IL‐1*β* and TNF‐*α*) were significantly decreased in TME. In contrast, Q11 significantly decreased M2‐like inflammatory factors and increased M1‐like inflammatory factors (Figure 7E,F). After IL‐4 (10 ng mL^−1^) and IL‐13 (10 ng mL^−1^) treatment for 24 h, primary microglia showed a typical amoeba‐like M2 microglia with fewer stubby projections and roundish cells. On the contrary, microglia in the low, medium, and high concentration groups showed branching or multiple protuberant M1‐like microglia (Figure 7G). In addition, Q11 significantly reduced Arg‐1 (M2 marker) and increased iNOS (M1 marker) expression (Figure 7H,I), evidenced by differential expression of cytokines at the protein level (Figure 7J) in the cell culture medium in primary microglia with or without Q11 treatment. These results suggest that inhibition of CYP2E1 by Q11 blocked microglia from polarizing to the M2‐type and promoted reprogramming to the M1‐type.

### 3.7 Q11 Regulated Microglia/Macrophages Reprogramming via PPAR‐*γ*‐STAT‐1/NF‐*κ*B/STAT‐3/STAT‐6 Pathway

**Figure 8:**
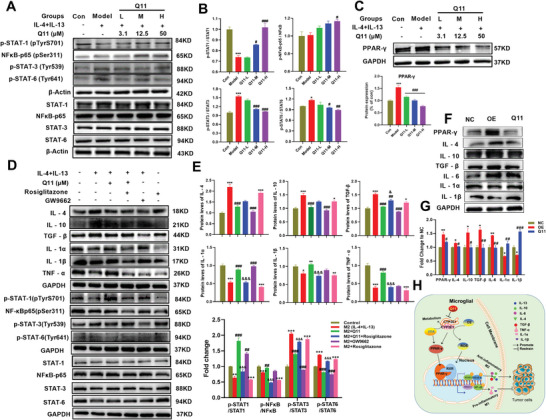
Q11 regulates microglia/macrophage reprogramming through the STAT‐1, NF‐*κ*B, STAT‐3, and STAT‐6 pathways in a PPAR‐*γ*‐dependent manner. A,B) Q11 regulated primary microglia reprogramming by promoting STAT‐1 and NF‐*κ*B activation and suppressing STAT‐3 and STAT‐6 activation. C) Effect of Q11 on protein of PPAR‐*γ* in primary microglia. D,E) Q11 regulation of microglia reprogramming at least in part in a PPAR‐*γ*‐dependent manner. F,G) Effect of upregulation of CYP2E1 on PPAR and inflammation of microglia in HMC‐3 cells. H) Schematic diagram of the molecular mechanisms underlying the inhibitory effects of Q11 on the regulation of microglia polarization in the TME of glioma. TME, tumor microenvironment. PPAR‐*γ*, peroxisome proliferator‐activated receptor‐*γ*. *n* = 3 for each group.

Phosphorylation of STAT‐1 (p‐STAT‐1) in primary microglia was significantly lower, while p‐STAT‐3 and p‐STAT‐6 significantly increased over that in the control group after treatment with IL‐4 and IL‐13 (**Figure** **8**A,B), suggesting that M1‐phenotypic signaling pathways were inhibited and M2‐phenotypic signaling pathways were activated after induction by IL‐4 and IL‐13. Compared with the IL‐4/IL‐13 group, p‐STAT‐1 and p‐NF‐*κ*B were significantly increased and p‐STAT‐3 and p‐STAT‐6 were significantly decreased in the Q11 medium‐ and high‐dose groups, suggesting that Q11 regulated microglia reprogramming by activating the STAT‐1 and NF‐*κ*B and inhibiting STAT‐3 and STAT‐6 signaling pathways. It has been reported that CYP2E1 is regulated by the peroxisome proliferator‐activated receptor *γ* (PPAR‐*γ*); therefore, we examined the effect of Q11 on PPAR‐*γ*. Indeed, the expression of PPAR‐*γ* in primary microglia was significantly increased after induction by IL‐4 and IL‐13, while Q11 significantly inhibited PPAR‐*γ* expression (Figure 8C). Rosiglitazone, an agonist of PPAR‐*γ*, completely blocked the effect of Q11. However, opposite to the effect of the agonist rosiglitazone, GW9662, an antagonist of PPAR*γ*, blocked the ability of rosiglitazone and downregulated the expression PPAR‐*γ* (Figure 8D,E). To confirm that CYP2E1 regulated microglial reprogramming through PPAR‐*γ*, we transfected CYP2E1 plasmids in HMC‐3 cells. Consistent with the results reported in the literature, upregulation of CYP2E1 in HMC‐3 cells significantly increased the expression of PPAR‐*γ*, accompanied by increased IL‐4, IL‐10, and TGF*β*, as well as decreased IL‐1*α* and IL‐1*β*, while Q11 significantly blocked the changes caused by CYP2E1 overexpression (Figure 8F,G). These findings reveal that Q11 regulates M/M*φ* reprogramming in a PPAR*γ*‐dependent manner. As a result, the possible molecular mechanisms underlying the inhibitory effects of Q11 on the growth of glioma by regulation of microglia reprogramming are summarized in Figure 8H.

## 4 Discussion

GBM, characterized by a large number of M2‐like tumor‐associated macrophages (TAMs) and inflammatory factors in TME,^[^ ^49^, ^50^ ^]^ lacks effective therapies, which leads to eventual tumor recurrence and poor survival.^[^ ^51^, ^53^ ^]^ Despite the abundance of preclinical trials conducted to identify novel, effective therapies for GBM, translation into actual clinical benefit has been rare.^[^ ^54^ ^]^ In this study, we demonstrated the crucial role of CYP2E1 in GBM by modulating M/M*φ* reprogramming and a newly developed CYP2E1 inhibitor, Q11, significantly suppressed the growth of GBM. More importantly, CYP2E1 inhibition could shifted the polarization of M/M*φ* in TME in a PPAR*γ*‐dependent manner reflected by an increase in M1 markers (e.g., IL‐1*α* and IL‐1*β*) and a decrease in M2 markers (e.g., IL‐4 and TGF‐*β*).

GBM is surrounded by a pool of pro‐tumorous inflammatory cytokines, chemokines and growth factors such as IL‐6, IL‐4, IL‐8, MCP‐1, IL‐10, IL‐13, and TGF‐*β*.^[^ ^55^ ^]^ In addition, high levels of inflammatory factors which contribute to tumor recurrence, growth, and TMZ resistance have been identified both in the conditioned media of several GBM cell lines and in the TME of GBM clinical samples.^[^ ^49^, ^56^ ^]^ For example, IL‐6 gene amplification in GBM tissue correlates with enhanced GBM aggressiveness and decreased patient survival.^[^ ^57^ ^]^ Although inflammation is closely related to the occurrence and progression of glioma, anti‐inflammatory therapy such as those targeting the COX axis have largely failed in large‐scale clinical trials for GBM.^[^ ^58^, ^59^ ^]^ This is most likely attributable to the heterogeneity of TME, diversity of inflammatory factors, and inadequacy of inflammatory targets.

CYP2E1, a member of the CYP450 family, mainly participates in the metabolism of endogenous substrates, including acetone and fatty acids^[^ ^29^, ^60^ ^]^ and activates exogenous toxic compounds and procarcinogens like ethanol, nicotine, and nitrosamine compounds as well as being involved in inflammation and oxidative stress. Recently, it was shown that CYP2E1 was associated with liver cancer,^[^ ^32^, ^34^, ^61^ ^]^ lung cancer,^[^ ^62^, ^63^ ^]^ and ovarian cancer^[^ ^35^ ^]^ by metabolizing and activating procarcinogens and inducing inflammation. Similarly, a possible association between CYP2E1 polymorphisms and the risk of glioma was demonstrated.^[^ ^64^ ^]^ However, there was no research on the precise mechanistic role of CYP2E1 in tumor progression, nor the testing of CYP2E1 as a target in clinical treatment of tumors.

CYP2E1 levels in brain are reported to be 25% of that in liver,^[^ ^29^, ^65^ ^]^ and activation or enhanced levels of CYP2E1 in the central nervous system (CNS) exacerbated neurological deficits and increased oxidative stress, inflammation, and neurodegeneration.^[^ ^66^, ^67^ ^]^ Furthermore, glial and neuronal cell cultures exhibited higher activity for CYP2E1 compared with the liver,^[^ ^68^ ^]^ implying that astrocytes are susceptibility to damage by inflammation and oxidative stress. Nonetheless, the role of CYP2E1 in the occurrence and progression of GBM is not completely understood.

In the current study, we found that CYP2E1 was remarkably higher in the glioma TME, and animal experiments further proved that CYP2E1 in TME was positively correlated with the severity of GBM. Systemic knockdown of *Cyp2e1* in rats and mice significantly inhibited the tumorigenesis and development of glioma, with tumor inhibition rates of 61.7% and 92.0% in knockout rats and mice, respectively, suggesting that CYP2E1 may be a potential effective therapeutic target for GBM. More importantly, phenotype observation of CYP2E1 gene knockout rats for 36 weeks showed that global *Cyp2e1*‐knockout rats did not suffer from any life‐threatening or life‐shortening conditions, suggesting that CYP2E1 as a GBM target has a good safety. These findings open a new window for the treatment of GBM.

Given the central role of CYP2E1 in inflammation and cancer progression, there is a strong rationale for the use of inhibitors of inflammation as therapeutic strategies for cancers. Reported inhibitors of CYP2E1 include chloridethiazole,^[^ ^69^, ^70^ ^]^ disulfiram,^[^ ^71^ ^]^ and phenylethylisothiocarbate (PEITC)^[^ ^72^ ^]^ and so on. Unfortunately, due to either low selectivity or weak inhibition, none have been developed for treating inflammation‐induced cancer, such as glioma. Based on this, we synthesized a novel inhibitor of CYP2E1 named Q11 with high selectivity and potent inhibition. Here, the pharmacokinetics of Q11 were determined; it showed good bioavailability and pharmacokinetic characteristics after oral administration. Importantly, both cyp2e1 gene knockout and CYP2E1 long‐term inhibition showed a good safety profile, as demonstrated by no adverse reactions noted in mice and rats with a *Cyp2e1* gene deletion, or with Q11 treatment in mice for 26 weeks and 12 weeks in rats.

Moreover, Q11 is one of the metabolites of the clinically used drug clomethiazole (IC_50_, 1.64 µm vs 39.3 µm), indicating stronger inhibition effect on CYP2E1 than clomethiazole. Q11 also has a higher selectivity. Chloromethiazole showed a significant central inhibitory effect at a dose that inhibited CYP2E1. However, Q11 only showed a slight inhibitory effect on CYP2A6 (IC_50_, 76.2 µm) and does not show a central inhibitory effect. These studies indicate good medicinal properties of Q11 with good efficacy and safety as an anti‐inflammatory agent for GBM.

Moreover, the blood brain barrier (BBB) prevents most cytotoxic chemotherapies and targeted agents from entering the brain and contributes to treatment failure. Hence, we first examined the pharmacokinetics of Q11 in SD rats. As expected, Q11 can penetrate the BBB to achieve a functionally relevant therapeutic concentration. Then we examined the effect of Q11 on orthotopic GBM allografts and xenografts, as orthotopic tumors are considered to better recapitulate the original conditions and particularly the microenvironments than do ectopic tumors, owing to the fact that tumor cells are directly implanted into their organ of origin. The in vivo anti‐glioma properties of Q11 in rats, mice, and nude mice intracranial xenografts showed that Q11 could both significantly inhibit GBM tumorigenesis and growth of GBM, and the inhibitory effect of Q11 was superior to that of TMZ. Importantly, Q11 also obviously improved tumor‐induced weight loss, and was less susceptible to drug resistance than TMZ since it acts on the TME. These results suggest that Q11 had high clinical transformation value and can be expected to provide a new strategy for the treatment of GBM.

Pro‐tumorous M2‐like TAMs plays an important role in the proliferation, invasion, and migration of GBM.^[^ ^73^, ^75^ ^]^ Ultimately, regulating TAM reprogramming is likely to be one of the treatment options for GBM. However, the success of developing therapeutic approaches to target TAM reprogramming for GBM therapy has yet been limited. It is known that peroxisome proliferator‐activated receptor *γ* (PPAR‐*γ*) expression is distinctly upregulated in MES GBM, and provides insight into PPAR‐*γ* as a potential therapeutic target for patients with MES GBM.^[^ ^76^ ^]^ Upregulation of CYP2E1 has been reported to increase PPAR‐*γ* expression,^[^ ^77^ ^]^ which is supported by our results. Moreover, in the current study we found that upregulation of CYP2E1 resulted in M/M*φ* reprogramming to M2‐like, which was manifested by activation of STAT‐3 and STAT‐6 signaling pathways and increased expression of pro‐tumorous M2‐like inflammatory factors such as IL‐10 and TGF‐*β*. On the contrary, inhibition of CYP2E1 by Q11 reprogrammed M/M*φ* to M1‐like, evidenced by inhibition of STAT‐3 and STAT‐6 signaling pathways and activation of STAT1 and NF‐*κ*B signaling pathways, and increased expression of anti‐tumorous M1‐like inflammatory factors such as IL‐1*α* and IL‐1*β*. Our studies have uncovered a new role for CYP2E1 and provided new insights into the pathogenesis of GBM.

This study mainly focuses on the role of CYP2E1 as a target for the prevention and treatment of glioma and CYP2E1 inhibitor in the prevention and treatment of glioma. However, it is necessary to study systematic selectivity of common transporters, highly expressed enzymes in the brain as well as drug combination studies from the point of view of new drug development. Given a large amount of work involved in the above research, a systematic regimen of medicinal properties of Q11 is necessary, which alone will be one of our next important research topics. Now pre‐trials of selectivity of CYPs highly expressed enzymes in the brain and combination regimen of Q11 combined with temozolomide, PD‐1, and so on is being explored and developed by our team.

In conclusion, we discovered a previously unknown pro‐tumorous mechanism in which the CYP2E1‐PPAR‐*γ*‐STAT‐1/NF‐*κ*B/STAT‐3/STAT‐6 axis induced GBM cell proliferation and inhibited apoptosis to fuel tumorigenesis and tumor growth. We also developed a potent inhibitor of CYP2E1, Q11, as an effective glioma anti‐inflammatory agent for the treatment of GBM, findings provide a perspective on anti‐inflammatory GBM therapy and offer a new potential treatment strategy for GBM.

## Conflict of Interest

The authors declare no conflict of interest.

## Author Contributions

G.H. and Y.F. contributed equally to this work. H.Q., G.H., and Y.F. designed and conducted most of the experiments, data analysis, and interpretation and wrote and edited the manuscript. H.X. conducted Synthesis and optimization of Q11 series compounds. G.W. and Y.R. conducted animal studies. F.G. inoculated GBM cells to prepare orthotopic models. Y.G. and C.Z. conducted the statistical analyses. Q.W. helped with some animal work. J.Q. performed histology sectioning and staining. N.G. and Q.W. developed the study concept, provided materials, and reviewed the manuscript. H.Q. directed the experimental design, oversaw the development of the study concept, and reviewed and edited the manuscript. All authors reviewed the manuscript and approved the content.

## Supporting information

Supporting Information

## Data Availability

The data that support the findings of this study are available from the corresponding author upon reasonable request.
