# Supplementary material for: Identification of Cytochrome P450 2E1 as a Novel Target in Glioma and Development of Its Inhibitor as an Anti‐Tumor Agent
Source: Adv Sci (Weinh). 2023 Jun 7;10(23):2301096. doi: 10.1002/advs.202301096 (PMC10427391; doi:10.1002/advs.202301096)
Supplement: Supplementary file 1 — Supporting Information [file ADVS-10-2301096-s001.pdf]

ADVANCED SCIENCE
Open Access

Supporting Information

for *Adv. Sci.*, DOI 10.1002/advs.202301096

Identification of Cytochrome P450 2E1 as a Novel Target in Glioma and Development of Its Inhibitor as an Anti-Tumor Agent

*Guiming Hu, Yan Fang, Haiwei Xu, Guanzhe Wang, Rui Yang, Fei Gao, Qingda Wei, Yuhan Gu, Cunzhen Zhang, Jinhuan Qiu, Na Gao, Qiang Wen and Hailing Qiao**

Supplementary Materials for

Identification of Cytochrome P450 2E1 as a novel target in glioma and development of its inhibitor as an anti-tumor agent

Guiming Hu[1, 2#], Yan Fang[1#], Guanzhe Wang[1], Rui Yang[1], Fei Gao[1], Qingda Wei[1], Yuhan Gu[1], Cunzhen Zhang[1], Jinhuan Qiu, Na Gao, Qiang Wen[1], Hailing Qiao[1*]

*Corresponding author. Email: qiaohl@zzu.edu.cn

This PDF file includes:

Figs. S1 to S4
Tables S1 to S2

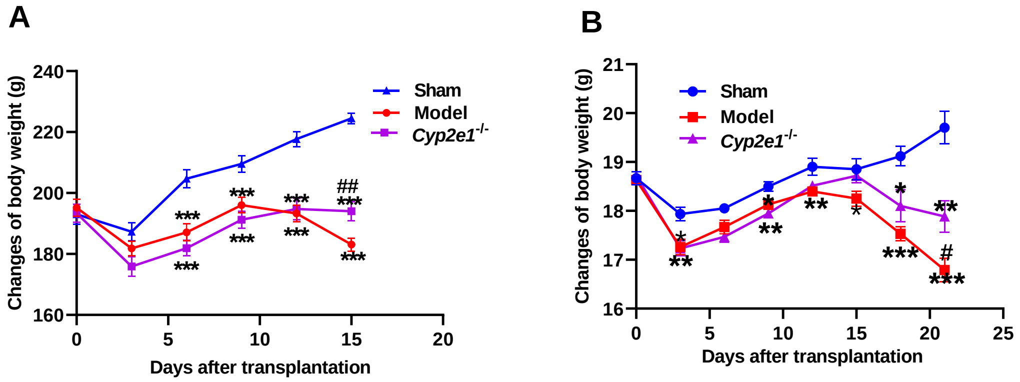

Fig. S1.Effect of *Cyp2e1*$^{-/-}$ on body weight in glioma-bearing murine animals. (A) Body weight of mouse bearing GL261 glioma cells. The experiment was lasted for 21 days and terminated when the first animal died. Mouse were weighed every 3 days to observe the dynamic change process of their body weight. (B) Body weight of C6 glioma-bearing rats. The experiment lasted for 15 days. Rats were weighed every 3 days to observe the dynamic change process of their body weight. Data are presented as Mean ± SD. Statistical analysis was performed by one-way ANOVA, Tukey's multiple comparisons test. Sham group (n = 10), Model group (n = 11), *Cyp2e1*$^{-/-}$ group (n = 9). *$P < 0.05$, **$P < 0.01$, ***$P < 0.001$ vs sham group, $^{\#}P < 0.05$, $^{\#\#}P < 0.01$ vs model group.

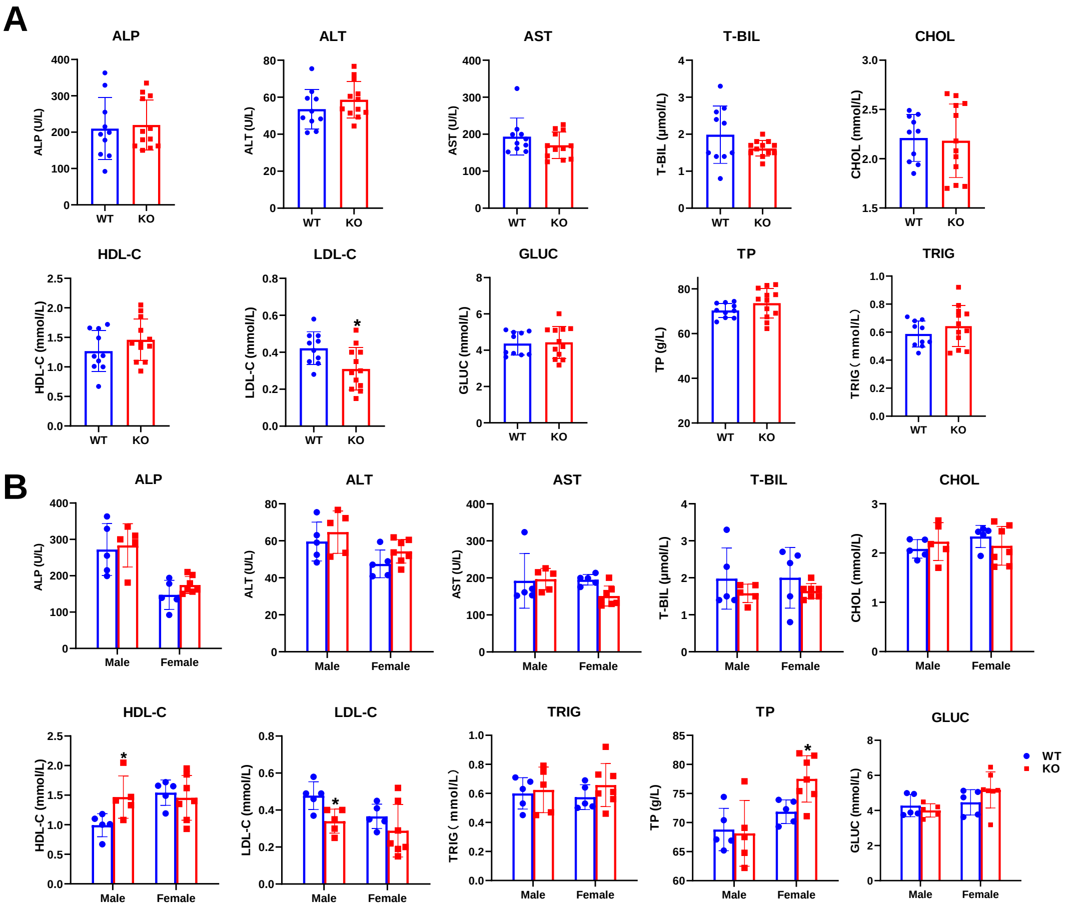

Fig. S2. Effects of *Cyp2e1* knockout on blood biochemical indexes at week 8. Serum biochemical indices between (A) *Cyp2e1* wild type (WT) and *Cyp2e1*$^{-/-}$ (KO) rats or (B) between genders. Data were shown as Mean ± SD of ten to twelve rats, *$P < 0.05$ vs WT. High-density lipoproteins-cholesterol (HDL-C), low-density lipoproteins-cholesterol (LDL-C), triglycerides (TRIG), cholesterol (CHOL), aspartate aminotransferase (AST), alanine amino transferase (ALT), alkaline phosphatase (AP), total proteins (TP), total bilirubin (T-BIL), glucose (GLUC) levels were compared between WT and KO rats. WT, wild type, KO, knockout of *Cyp2e1*.

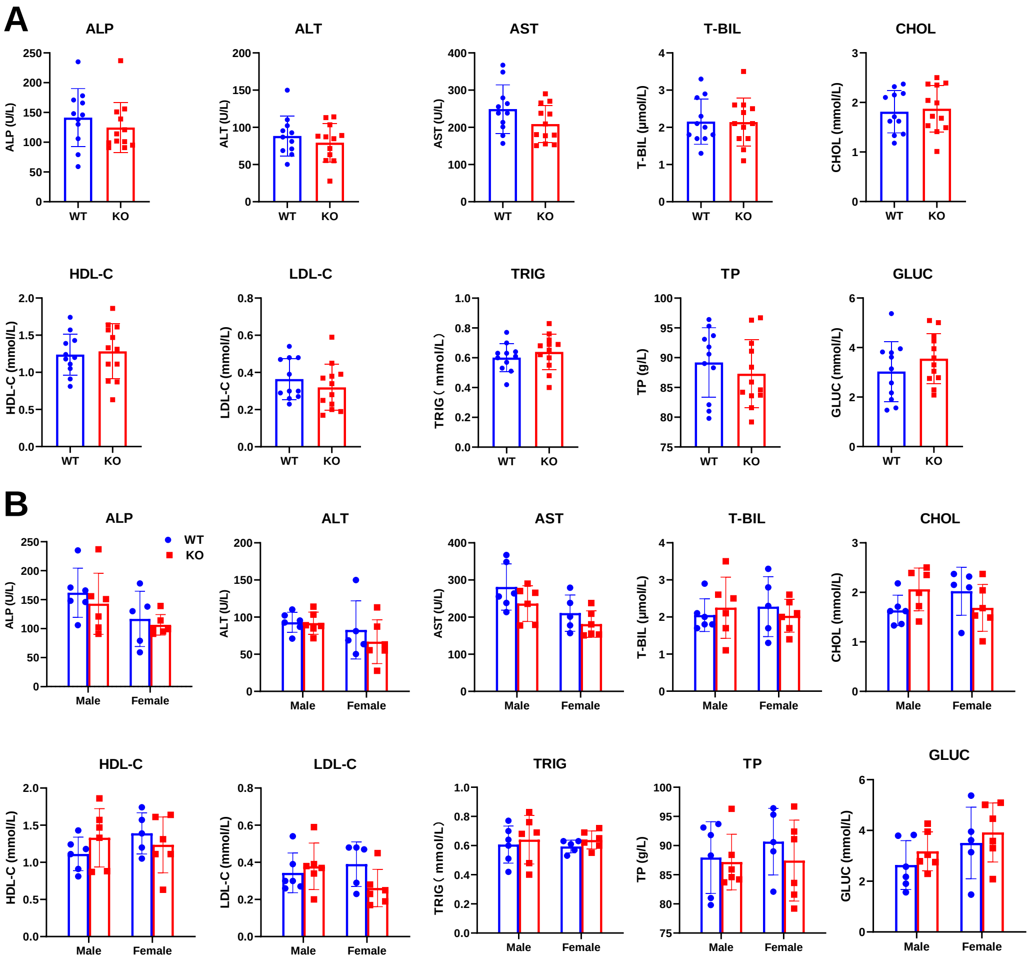

Fig. S3. Effects of *Cyp2e1* knockout on blood biochemical indexes at week 36. Serum biochemical indices between (A) *Cyp2e1* wild type (WT) and *Cyp2e1*$^{-/-}$ (KO) rats or (B) between genders. Data were shown as Mean ± SD of ten to twelve rats, *P < 0.05 vs WT. High-density lipoproteins-cholesterol (HDL-C), low-density lipoproteins-cholesterol (LDL-C), triglycerides (TRIG), cholesterol (CHOL), aspartate aminotransferase (AST), alanine amino transferase (ALT),

alkaline phosphatase (AP), total proteins (TP), total bilirubin (T-BIL), glucose (GLUC) levels were compared between WT and KO rats. WT, wild type, KO, knockout of *Cyp2e1*.

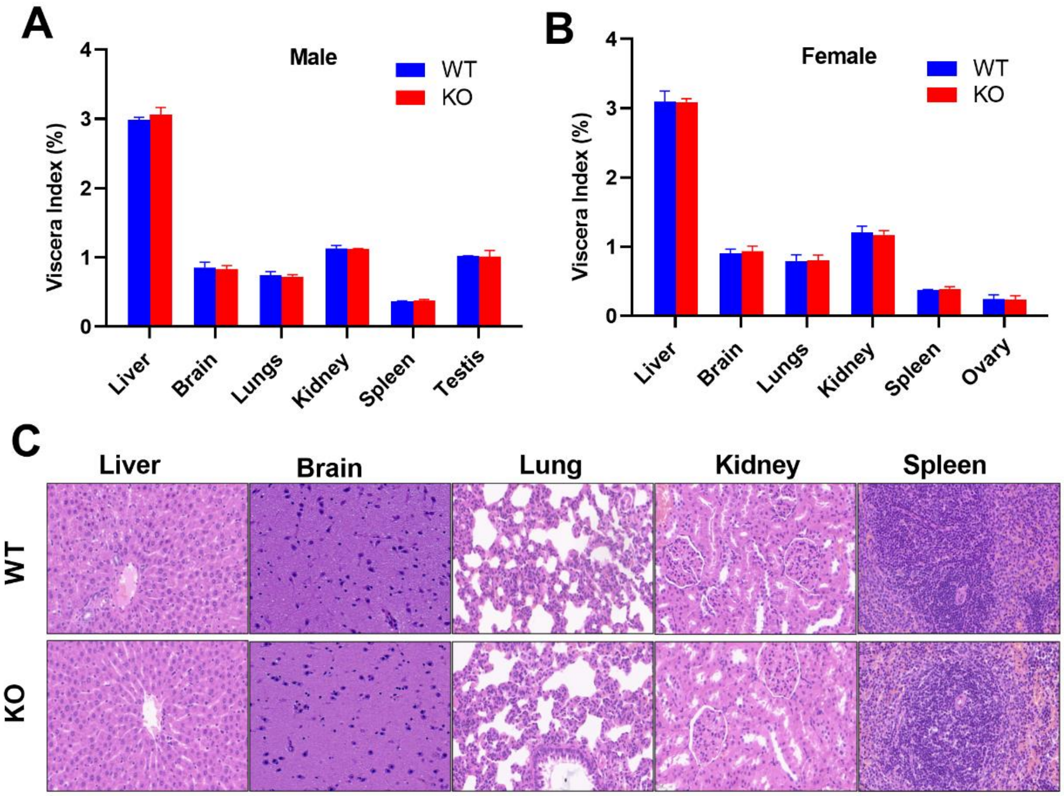

Fig. S4. Effects of *Cyp2e1* knockout on organ pathology in rats. (A) Organ index of male rats. (B) Organ index of female rats. (C) Organ morphologic.

Table S1. Primers for real-time PCR

Genes	Forward primer (5’-3’)	Reverse primer (5’-3’)
**Human primers**		
GAPDH	GGAAGCTTGTCATCAATGGAAATC	TGATGACCCTTTTGGCTCCC
IL-4	GGTCTCACCTCCCAACTGCTTC	AGTCTTCTGCTCTGTGAGGCTGTT
IL-1α	AACCAGTGCTGCTGAAGGAGAT	CAATAAACAAGTTTGGATGGGC
IL-6	TTCGGTCCAGTTGCCTTCTC	AGTGCCTCTTTGCTGCTTTCA
IL-10	GCTGAGAACCAAGACCCAGACAT	GCATTCTTCACCTGCTCCACG
IL-13	GTCTCAGCTGGGCAGTTTTCC	TCCTTTACAAACTGGGCCACC
IL-12α	TAAAACCAGCACAGTGGAGGC	GGCCAGGCAACTCCCATTAG
IL-17α	TTGGTGTCACTGCTACTGCTGCT	TGGTAGTCCACGTTCCCATCAG
TNF-α	TCTACTCCCAGGTCCTCTTCAAG	GGAAGACCCCTCCCAGATAGA
TGF-β	CCCACAACGAAATCTATGACAAG	GCTGAGGTATCGCCAGGAAT
PPARα	CCTCGGTGACTTATCCTGTGGT	GACATCCCGACAGAAAGGCAC
PPARβ	CAAGATTCAGAAGAAGAACCGCA	ACAATGTCTCGATGTCGTGGATC
PPARγ	TGTCTCATAATGCCATCAGGTTTG	CTCGCCTTTGCTTTGGTCAG
APOE	CTGCGTTGCTGGTCACATTC	GTAATCCCAAAAGCGACCCAG
HMGCR	AAAGGAGGCATTTGACAGCAC	AGCAGCAGGTTTCTTGTCAGTAC
SREBP2	AGGCAGGCTTTGAAGACGAA	TGCACATTCAGCCAGGTTCAC
CYP46A1	AGGCAGTGAAACTTATGTTGGAGG	CAGCAATGAAGAAGGTGACGAA
TREM2	CGGCTGCTCATCTTACTCTTTGTC	AGGGTATCGTCTGTGATGGCT
ABCG1	AGAAGGATGAAGGCAGAAGGGA	GTGGATGGTGCAAATGATGGAG
ABCA1	AGCGGGTCAGCAAAGCAA	TGTGGCAGGGACAACGTAATT

ACAT1	AAGTAAAGCAGCATGGGAAGC	GCCAGTGGTGTAACATTGAGCC
CYP2E1	TGCCTGCTCGTGGAAATG	GGGTGATGAACCGCTGAA
VEGF	TCACCAAGGCCAGCACATAG	GAGGCTCCAGGGCATTAGAC
CXCL12	TGAGCTACAGATGCCCATGC	GCACACTTGTCTGTTGTTGTTCTTC
**Mouse primers**		
GAPDH	CCTCGTCCCGTAGACAAAATG	TGAGGTCAATGAAGGGGTCGT
IL-4	AAACTCCATGCTTGAAGAAGAACTC	TGATGCTCTTTAGGCTTTCCAG
IL-10	CACTGCTATGCTGCCTGCTCT	GTCGGTTAGCAGTATGTTGTCCAG
IL12 α	TCACGCTACCTCCTCTTTTTGG	TGGTTTGGTCCCGTGTGATGT
IL-13	TCTTGCTTGCCTTGGTGGTC	TGCAATATCCTCTGGGTCCTGT
IL17 α	TCCACCGCAATGAAGACCCT	CATGTGGTGGTCCAGCTTTCC
TNF- α	AGACCCTCACACTCACAAACCA	CTTTGAGATCCATGCCGTTG
IL-1 α	CGGGTGACAGTATCAGCAACGT	TGACAAACTTCTGCCTGACGAG
TGF- β	GCTGAACCAAGGAGACGGAATA	GGCTGATCCCGTTGATTTCC
IL-1 β	TCGCAGCAGCACATCAACAAGAG	AGGTCCACGGGAAAGACACAGG
PPAR α	GAGAATCCACGAAGCCTACCTG	GACCTCTGCCTCTTTGTCTTCG
PPAR β	CAGCCACAACGCACCCTTT	GTCACCTGGTCATTGAGGAAGA
PPAR γ	GACCACTCGCATTCCTTTGACA	ATCGCACTTTGGTATTCTTGGA
HMGCR	TTTGCTCGTCTACAGAAACTCCAC	CTCACCACCTTGGCTGGAATG
ACAT1	TGAGCAGAGGAGCAACACCATA	TGGGAGTAATCTCACTGGCAAAC
ABCA1	AAGTTTCTGCCCTCTGTGGTCTA	TGGGTCGGGAGATGAGATGTG
APOE	CGTGCTGTTGGTCACATTGC	CATCAGTGCCGTCAGTTCTTGT
ABCG1	TTGTGCTGTTCGCTGCTCTG	GTCACGGGACCCACAAATGT
SREBP2	TCTGAGGAAGGCCATTGATTACA	CAAGTCCACATCACTGTCCACCA
CYP46A1	TGGTCCTGTTGTAAGAGTCAATGTC	AAACAGTCTCTCCCCAAACACAG
TREM2	AAGAGACCTCCTTCCCACCC	GAATTCTCTCACGTACCTCCGG
CYP2E1	AGGCTGTCAAGGAGGTGCTAC	GCACAGCCAATCAGAAAGGTAG
VEGF	GAGCGTTCACTGTGAGCCTTGT	TTAACTCAAGCTGCCTCGCCT
BDNF	TATTAGCGAGTGGGTCACAGCG	TACGATTGGGTAGTTCGGCATT
CXCL12	AGTCAGCCTGAGCTACCGATG	TTCTTCAGCCGTGCAACAATC

Table S2 Structure-activity relationship of Q11

IC_{50} values of the training set compounds

Structure Optimization	Compound	Structure	IC_{50} (μM)
Lead compound	Q11	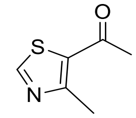	1.64
Bioelectronic isostere replacement of the thiazole group of Q11	SMI1	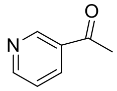	>100
	SMI2	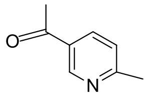	>100
	SMI3	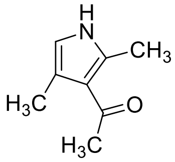	>100
	SMI4	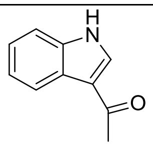	>100
	SMI5	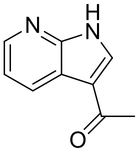	>100
	SMI6	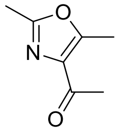	>100
	SMI7	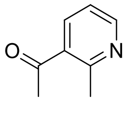	>100

	SMI8	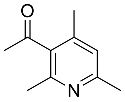	>100
	SMI9	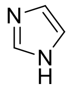	49.8
	SMI10	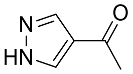	58.3
	SMI11	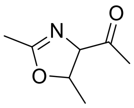	>100
	SMI12	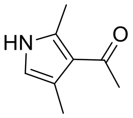	>100
	SMI13	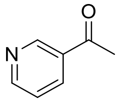	>100
	SMI14	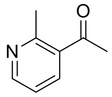	>100
	SMI15	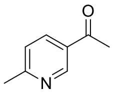	>100
	SMI16	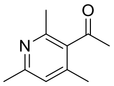	>100

	SMI17	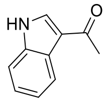	>100
	SMI18	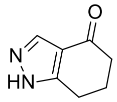	33.8
	SMI19	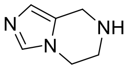	>100
	SMI20	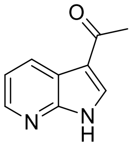	>100
	SMI21	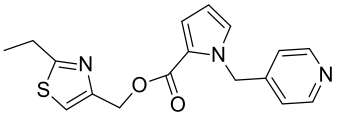	7.99
	SMI22	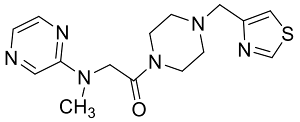	>100
	SMI23	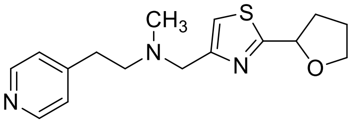	>100
	SMI24	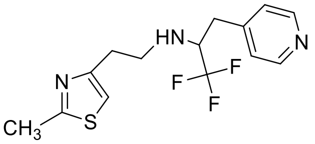	>100
	SMI25	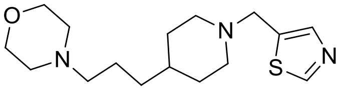	>100

Similarity search base on Q11	SMI26	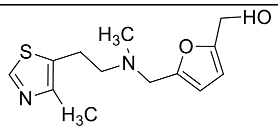	>100
Modification of acetyl group on Q11	SMI27	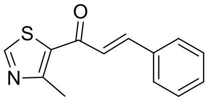	>100
	SMI28	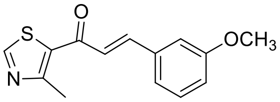	>100
	SMI29	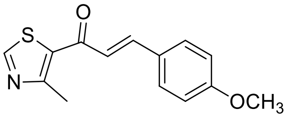	>100
	SMI30	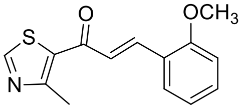	>100
	SMI31	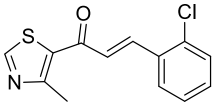	>100
	SMI32	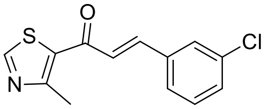	>100
	SMI33	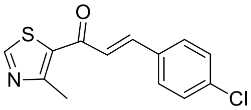	>100
	SMI34	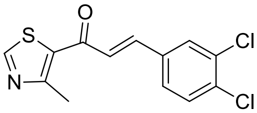	>100
	SMI35	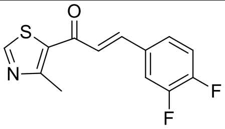	>100
	SMI36	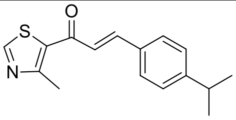	>100

	SMI37	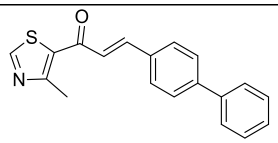	>100
	SMI38	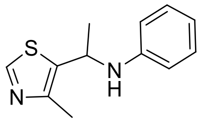	>100
	SMI39	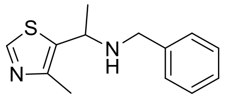	>100
	SMI40	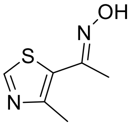	>100
	SMI41	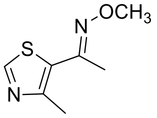	17.0
	SMI42	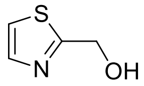	>100
	SMI43	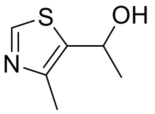	>100
	SMI44	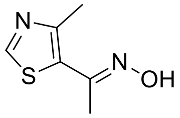	>100
	SMI45	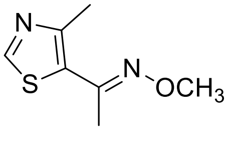	17.<br>0

	SMI46	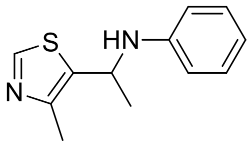	>100
	SMI47	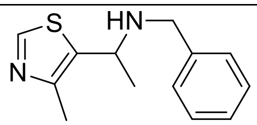	>100
	SMI48	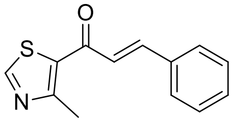	>100
	SMI49	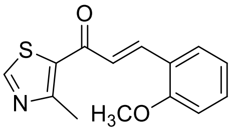	>100
	SMI50	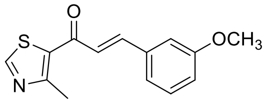	>100
	SMI51	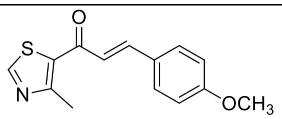	>100
	SMI52	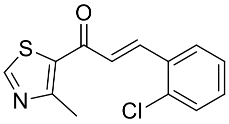	>100
	SMI53	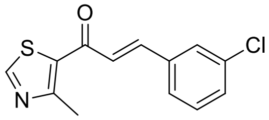	>100
	SMI54	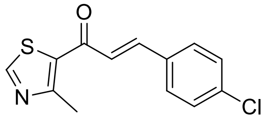	>100

	SMI55	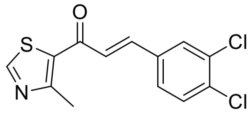	>100
	SMI56	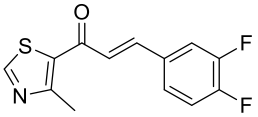	>100
	SMI57	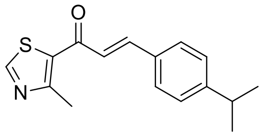	>100
	SMI58	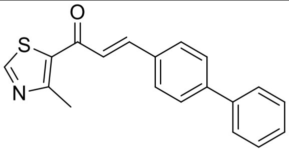	>100
	SMI59	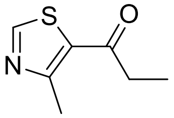	>100
	SMI60	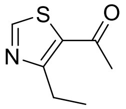	>100
Modification of methyl group or hydrogen on Q11	SMI61	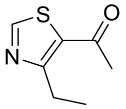	>100
	SMI62	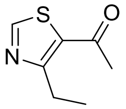	>100
